# Neurite Outgrowth in PC12 Cells Stimulated by Components from *Dendranthema* × *grandiflorum* cv. “Mottenohoka” Is Enhanced by Suppressing Phosphorylation of p38MAPK

**DOI:** 10.1155/2013/403503

**Published:** 2013-03-10

**Authors:** Atsuyoshi Nishina, Hirokazu Kimura, Hiroyuki Tsukagoshi, Kunihisa Kozawa, Mamoru Koketsu, Masayuki Ninomiya, Shoei Furukawa

**Affiliations:** ^1^Yonezawa Women's Junior College, 6-15-1 Tohrimachi, Yonezawa, Yamagata 992-0025, Japan; ^2^Gunma Prefectural Institute of Public Health and Environmental Sciences, 378 Kamioki, Maebashi, Gunma 371-0052, Japan; ^3^Department of Materials Science and Technology, Faculty of Engineering, Gifu University, Gifu 501-1193, Japan; ^4^Laboratory of Molecular Biology, Department of Biofunctional Analysis, Gifu Pharmaceutical University, Daigaku-nishi, Gifu 501-1196, Japan

## Abstract

Components from *Dendranthema* × *grandiflorum* cv. “Mottenohoka” that promote neurite outgrowth of PC12 cells were identified and the mechanism of neurite outgrowth stimulated by isolated components was studied. Components that promoted the phosphorylation of extracellular signal-regulated kinase 1/2 (ERK 1/2) of PC12 cells were isolated. From various structural analyses, the active components were identified as acacetin and luteolin. The effects of acacetin or luteolin on PC12 cells were evaluated by electro-blotting and immunostaining. Slight neurite outgrowth in PC12 cells was observed within 2 days of culture after stimulation by luteolin or acacetin. However, NGF-stimulation induced remarkable neurite outgrowth in comparison. Neurite outgrowth by luteolin or acacetin was significantly enhanced by pretreatment with SB203580 (a p38MAPK inhibitor). The results of this study into the phosphorylation of ERK 1/2 and p38MAPK by flavonoids suggest that the inhibition of p38MAPK phosphorylation may effectively enhance neurite outgrowth.

## 1. Introduction

 Spring cherry blossoms and the chrysanthemums of autumn are typical ornamental and edible flowers of Japan. *Dendranthema × grandiflorum* cv. “Mottenohoka,” a distinctive edible chrysanthemum with a beautiful color, pleasant aroma, and crunchy texture, is a special product of north-eastern Japan and has traditionally been eaten as a delicacy around the country. The pharmacological effects of *Chrysanthemum morifolium*, including Mottenohoka, are various and include sleep induction [1], protection of brain cells against ischemia [2], toxicity to cancer cells [3], suppression of intercellular adhesion molecule-1 and E-selectin expression [4], protection of neuroblastoma SH-SY5Y cells [5], cardiac protection against ischemia [6], anti-HIV effect [7, 8], and antimutagenicity [9]. Components of *Chrysanthemum morifolium *that may be associated with these pharmacological effects are luteolin and apigenin [10, 11], acidic polysaccharide [3, 12], dicaffeoylquinic acids [13], chlorogenic acid, 3,5-*O*-dicaffeoylquinic acid, 3′,4′,5-trihydroxyflavanone 7-*O*-glucuronide [14], apigenin 7-*O*-*β*-d-(4′-caffeoyl)glucuronide and apigenin 7-*O*-*β*-d-glucuronide [7], acacetin, apigenin, luteolin and quercetin [9], and acacetin 7-*O*-*β*-d-galactopyranoside [8].

We tested the effects of extracts from various specialty vegetable products of northeastern Japan on the intracellular signaling of PC12 cells and found that Mottenohoka extracts increased the level of phosphorylated extracellular signal-regulated kinases 1/2 (ERK 1/2). It was important to determine whether the Ras/ERK 1/2 pathway is involved in ERK 1/2 phosphorylation induced by Mottenohoka extracts because the activation of the pathway leads to neuritogenesis followed by neuronal differentiation of PC12 cells. Thus, the substances that can activate Ras/ERK 1/2 might be promising for protection against or therapy for some neurological disorders with suspected neurotrophic factor involvement, such as depression or Alzheimer's disease. These considerations prompted us here to identify the components from Mottenohoka that activate mitogen-activated protein kinase (MAPK) of PC12 cells and to clarify the intracellular signals so that their possible therapeutic use in neurological disorders may be explored.

## 2. Materials and Methods

### 2.1. Reagents

3-(4,5-Dimethylthiazol-2-y)-2,5-diphenyl-2H-tetrazolium bromide (MTT) was purchased from Sigma-Aldrich (St. Louis, MO, USA). Hexane, chloroform, and methanol were obtained from Wako Pure Chemical Industries (Osaka, Japan). A specific inhibitor for tyrosine kinase of epidermal growth factor receptor (EGFR) (AG1478), an inhibitor of Trk-type tyrosine kinases (K252a), MEK inhibitor (U0126), and p38 mitogen activated protein kinase (p38MAPK) inhibitor (SB203580) were purchased from Calbiochem (San Diego, CA, USA) or Nacalai Tesque Inc. (Kyoto, Japan).

### 2.2. Solvent Fractionation

**Figure 1:**
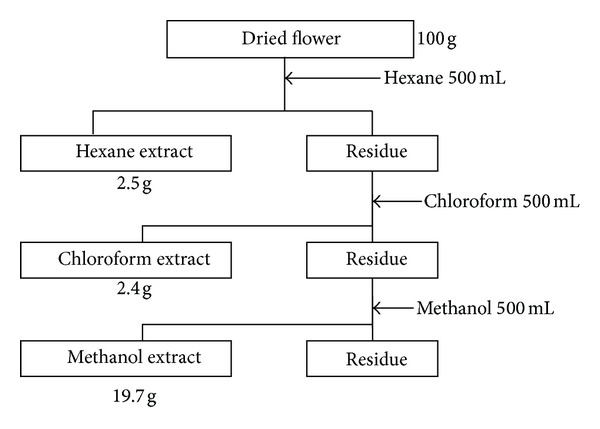
Scheme for solvent fractionation of extracts of *Dendranthema × grandiflorum* cv. “Mottenohoka.”

Raw Mottenohoka was purchased commercially from Yamagata prefecture and the components were fractionated according to a flow sheet (Figure 1). In brief, Mottenohoka (100 g) was dried, powdered, and immersed in 500 mL of hexane for 24 h at room temperature. The solvent containing the extracts was filtrated through a filter paper (5C; Whatman, Brentford, UK) and the filtrate was evaporated to dryness (hexane extract; 2.5 g). The residue was then stirred in 500 mL of chloroform at room temperature for 24 h and filtrated, and the filtrate was evaporated to dryness (chloroform extract; 2.4 g). Methanol extract (19.7 g) was prepared in a similar manner.

### 2.3. Refining of Active Components

Methanol extract (10 g) was applied for Sephadex LH-20 column chromatography (CC) and eluted with methanol into 100 fractions. The activity of each fraction was evaluated in cultured PC12 cells. An aliquot of the active fractions was injected into preparative high performance liquid chromatography equipped with a Develosil ODS 60–10 column (20 × 250 mm, 10 *μ*m particle size; Nomura Chemical Co., Ltd., Aichi, Japan), a PU-800 pump (JASCO, Tokyo Japan), and an evaporative light scattering detector (SEDEX model 75; Sedere Inc., Cranbury, NJ, USA). The column was run using a water : methanol : trifluoroacetic acid = 30 : 70 : 0.1 (v/v/v) mobile phase at a flow rate of 10 mL/min. The eluted substances were collected separately by referring to the signal detected by the evaporative light scattering detector.

### 2.4. Analytical Instrument of Active Components

^1^H (400 MHz) NMR spectra were recorded with a JEOL ECX 400 spectrometer, using tetramethylsilane as an internal standard.

### 2.5. Cell Culture

In the present study, we used rat pheochromocytoma cell line PC12 because previous reports suggested the suitability of this cell line as a model for studying mechanisms of neuronal differentiation including neuritogenesis [15]. The cells were cultured as described previously [16]. In brief, cells were maintained in Dulbecco's modified Eagle's medium (DMEM; Sigma) supplemented with 10% heat-inactivated horse serum (HS; Gibco BRL, Grand Island, NY, USA) and 5% heat-inactivated fetal bovine serum (FBS, Sanko Junyaku Co., Ltd., Tokyo, Japan) (serum-containing medium), or in DMEM supplemented with 1% bovine serum albumin (BSA) (serum-free medium). Photographs of cells were taken under phase-contrast observation at 100x magnification.

### 2.6. Measurement of Cytotoxicity

PC12 cells were cultured on collagen-coated 96-well plates (2 × 10^6^ cells/well) in serum-containing medium for 2 days at 37°C in an atmosphere of 95% air/5% CO_2_. Culture medium was replaced with 50 *μ*L of serum-free medium containing each test agent after washing with PBS, and the cells were cultured with each test agent over a range of days. Cell viability was determined by the 3-(4,5-dimethyl-2-thiazolyl)-2,5-diphenyl-*2H*-tetrazolium bromide (MTT) reduction assay [17]. The cells were incubated with MTT (0.25 ng/mL; final concentration) for 2 h and the reaction was stopped by adding 50 *μ*L of 50% (v/v) dimethylformamide (DMFA) containing 20% (w/v) sodium dodecyl sulfate (SDS). The amount of MTT formazan product was determined photometrically using a microplate reader (Ultrospec Visible Plate Reader II, Amersham Biosciences, Tokyo, Japan) by measuring the absorbance at 562 nm with a reference wavelength of 630 nm. The number of cells in each well was calculated on the basis of a known relationship between absorbance and cell number.

### 2.7. Detection of Phosphorylated Proteins

Each test compound was suspended in serum-free medium and sonicated until fully emulsified. PC12 cells were seeded at 2 × 10^6^ cells/well onto collagen-coated 6-well plates (Corning Life Sciences, Lowell, MA, USA) supplemented with the serum-containing medium and cultured for 2 days at 37°C in an atmosphere of 95% air/5% CO_2_. The cells were then washed with phosphate-buffered saline (PBS) and incubated with the above mentioned serum-free culture medium containing the various agents for 10 min at 37°C. The culture plates were then placed on ice and each well was washed with 3 mL of 2 mM Tris-HCl buffer (pH 8.0) containing 0.33 M NaF and 6.25 M Na_3_VO_4_, and subsequently lysed with 150 *μ*L of 20 mM Tris-HCl buffer (pH 8.0) containing 150 mM NaCl, 2 mM EDTA, 1% Nonidet P-40 (w/v), 1% sodium deoxycholate (w/v), 0.1% SDS (w/v), 50 mM NaF, 0.1% aprotinin (w/v), 0.1% leupeptin (w/v), 1 mM Na_3_VO_4_, and 1 mM phenylmethylsulfonylfluoride (PMSF). Cell lysates were collected by using a cell scraper and were centrifuged at 15000 ×g for 30 min at 4°C. The supernatant was collected and the overall protein concentration was determined by a BCA Protein Assay Reagent Kit (Pierce, Rockford, IL, USA) with BSA as the standard.

Supernatant fluids containing proteins (20 *μ*g) were mixed with lithium dodecyl sulfate (LDS) sample buffer (Invitrogen Corp, Carlsbad, CA, USA) and were incubated for 5 min at 80°C. Proteins in the samples were separated on SDS-polyacrylamide gel electrophoresis, and the proteins in gels were electroblotted onto polyvinylidene fluoride (PVDF) filters (Fluorotrans membrane W, 0.2 *μ*m; Nihon Genetics, Tokyo, Japan). Immunoblotting analysis was performed by using monoclonal antibodies against p44/42 ERK, phospho-p44/42 ERK, p38MAPK, phospho-p38MAPK, stress-activated protein kinase/Jun amino-terminal kinase (JNK/SAPK), phospho-JNK/SAPK, Akt, phospho-Akt, neurofilament-M, or *β*-actin (Cell Signaling Technology, Lake Placid, NY, USA) as the primary antibodies, followed by reaction with horseradish peroxidase-conjugated anti-rabbit or anti-mouse IgG antibodies from Promega Corporation (Fitchburg, WI, USA) as the secondary antibody. The blots were developed by the enhanced chemiluminescence method (Hyperfilm ECL-plus, Amersham Biosciences Corp., Piscataway, NJ, USA).

Image J was used for densitometry calculation [18]. To evaluate the level of phosphorylated protein, the ratio of intensity of phosphorylated protein/intensity of total protein was measured. When images obtained from multiple experiments were compared, the common sample (control) was always analyzed in all experiments and the control values were used to correct the value of samples on Image J.

### 2.8. Treatment with Specific Inhibitors

Each inhibitor was used at a final concentration of 1 *μ*M (K252a, AG1478, and SB203580) or 10 *μ*M (U0126) in serum-containing medium from a preliminary evaluation test for dose responsiveness between the concentration of inhibitor and the level of phosphorylated protein. PC12 cells were seeded at 2 × 10^6^ cells/well supplemented with serum-containing medium and precultured for 2 days at 37°C in an atmosphere of 95% air/5% CO_2_. Cells were preincubated for 3 h in the serum-free medium containing each inhibitor, washed with PBS, and incubated with the medium containing the corresponding active substance for 10 min at 37°C. Cells were then collected and the cell lysates were used for immunoblot analyses of phosphorylation or expression of proteins.

## 3. Results

### 3.1. Yield and Cytotoxicity of Each Fraction

The method of solvent fractionation is shown in Figure 1. Hexane extract (2.5 g), chloroform extract (2.4 g), and methanol extract (19.7 g) were obtained from 100 g of dried Mottenohoka. None of the extracts were cytotoxic to PC12 cells at less than 100 *μ*g/mL (data not shown).

### 3.2. ERK 1/2 Activation by Solvent Extracts

**Figure 2:**
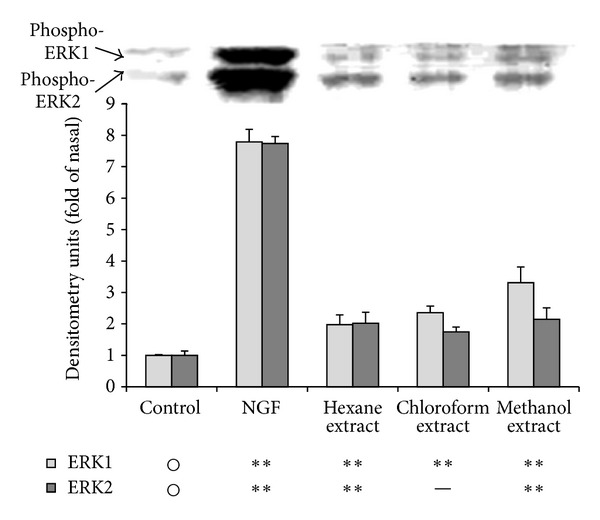
Ability of each fraction to activate extracellular signal-regulated kinase 1/2 (ERK 1/2) of PC12 cells.

The levels of phosphorylated ERK 1/2 of PC12 cells after treatment with solvent extracts of Mottenohoka are shown in Figure 2. Methanol extract exhibited the most potent relative effect among the three types of solvent extract, thus we decided to isolate the active components.

### 3.3. Isolation of Active Components by Chromatography

Methanol extract was applied to a Sephadex LH-20 column equilibrated with methanol and eluted with methanol as the mobile phase. The fractions were collected in the order of elution (no. 1 to 100). Enhanced ERK 1/2 activation was found in fractions no. 40 to 100. The active fractions were pooled, lyophilized, and applied to an ODS column for reverse phase preparative chromatography. We obtained three kinds of active components (components **1**–**3**) from the mixture of fractions no. 40 to 100.

### 3.4. Structural Determination of Isolates

The ^1^H NMR of component **1** showed seven signals. A set of three double doublets was observed at *δ*_H_ 5.40, 3.14, and 2.72, which are characteristic signals of the flavanone skeleton. Two singlets at *δ*_H_ 5.97 and 5.95 indicated that this component was 5,7-dihydroxyflavanone. In addition, the signals in the downfield region (*δ*_H_ 7.04 and 6.88) showed the existence of a 1,3,5-trisubstituted aromatic ring. These findings led us to determine that the structure of compound **1** was 3′,5,5′,7-tetrahydroxyflavanone.

**Figure 3:**
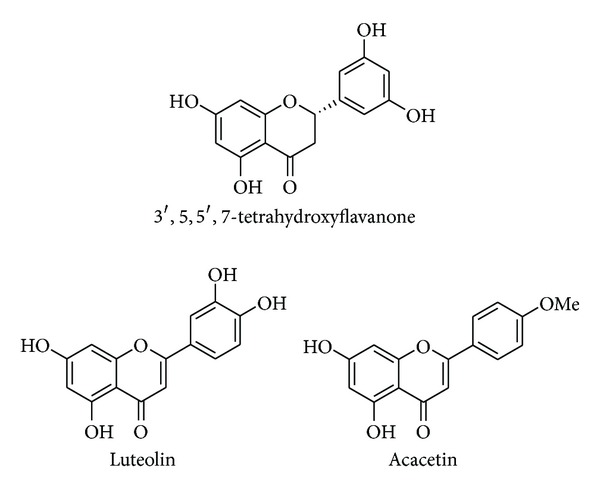
Chemical structures of isolates.

The ^1^H NMR of component **2** showed the characteristic signals of 5,7-dihydroxyflavone; the singlet at *δ*_H_ 6.59 was assigned to the H-3 position of the flavone skeleton. In addition, three signals (*δ*_H_ 7.52, 7.48, and 7.01) showed the existence of a 1,3,4-trisubstituted aromatic ring. Thus, the structure of component **2** was established as luteolin. The ^1^H NMR of component **3** exhibited signals similar to those of component **2**. The spectrum clearly showed the signals of a *para*-substituted aromatic ring (*δ*_H_ 8.04 and 7.14) and a methoxy group (*δ*_H_ 3.92). The structure of component **3** was established as acacetin (Figure 3).

3′,5,5′,7*-Tetrahydroxyflavanone.* ^1^H NMR (acetone-*d*_6_, 400 MHz): *δ* 7.04 (1H, s, H-4′), 6.88 (2H, s, H-2′ and H-6′), 5.97 (1H, s, H-8), 5.95 (1H, s, H-6), 5.40 (1H, dd, *J* = 13.3 and 3.2 Hz, H-2), 3.14 (1H, dd, *J* = 17.4 and 13.3 Hz, H-3*α*), 2.72 (1H, dd, *J* = 17.4 and 3.2 Hz, H-3*β*).

*Luteolin*. ^1^H NMR (acetone-*d*_6_, 400 MHz): *δ* 7.52 (1H, s, H-2′), 7.48 (1H, d, *J* = 8.7 Hz, H-6′), 7.01 (1 H, d, *J* = 8.7 Hz, H-5′), 6.59 (1H, s, H-3), 6.54 (1H, s, H-8), 6.26 (1H, s, H-6).

*Acacetin*. ^1^H NMR (acetone-*d*_6_, 400 MHz): *δ* 8.04 (2H, d, *J* = 8.7 Hz, H-2′ and H-6′), 7.14 (2H, d, *J* = 8.7 Hz, H-3′ and H-5′), 6.69 (1H, s, H-3), 6.57 (1H, s, H-8), 6.28 (1H, s, H-5), 3.92 (3H, s, OMe).

### 3.5. MAPK Activation in PC12 Cells by the Isolated Components

**Figure 4:**
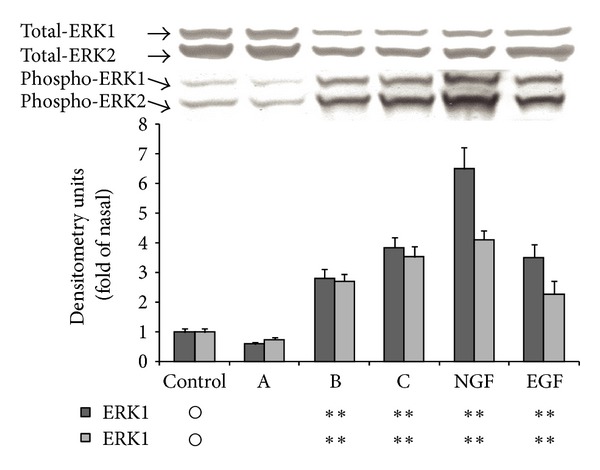
Effects of 3′,5,5′,7-tetrahydroxyflavanone (A), luteolin (B), acacetin (C), NGF, or EGF on the level of phosphorylated ERK 1/2 of PC12 cells. PC12 cells were cultured in serum-containing medium for 2 days. They were treated or not treated with A, B, C, NGF, or EGF for 10 min. The level of phosphorylated ERK 1/2 of each sample was evaluated by immunoblotting. The intensity of each band of ERK 1/2 was measured densitometrically by Image J. Values are expressed as the ratio of phospho-ERK 1/2 to total- ERK 1/2, and the mean ± SEM of the values of three separate experiments are shown. Significant differences of the values from the value of the corresponding control group were determined by ANOVA, followed by Dunnett's test. Control group is signified as ○. *: significance difference at *P* < 0.1; **: significant difference at *P* < 0.01; —: no significant difference.

**Figure 5:**
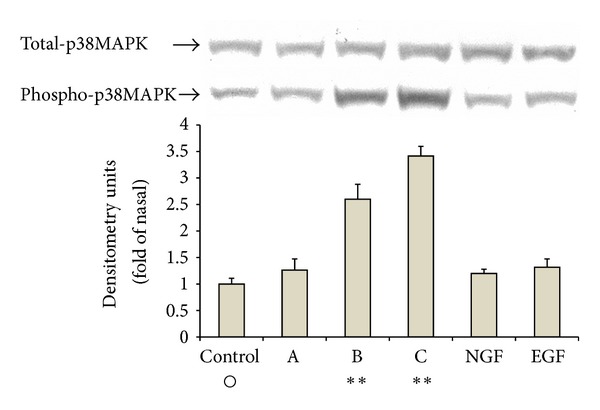
Effects of 3′,5,5′,7-tetrahydroxyflavanone (A), luteolin (B), acacetin (C), NGF, or EGF on the level of phosphorylated p38 mitogen activated protein kinase (p38MAPK) of PC12 cells. Experimental procedures and methods for statistical analysis are the same as those shown in the legend of Figure 4.

**Figure 6:**
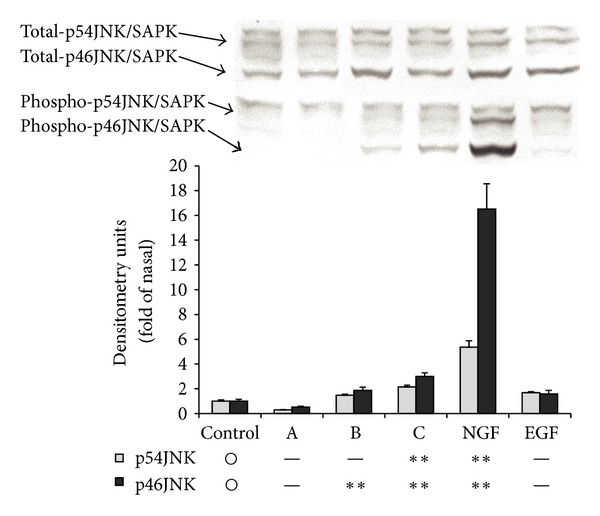
Effects of 3′,5,5′,7-tetrahydroxyflavanone (A), luteolin (B), acacetin (C), NGF, or EGF on the level of phosphorylated stress-activated protein kinase/Jun amino-terminal kinase (JNK/SAPK) of PC12 cells. Experimental procedures and methods for statistical analysis are the same as those shown in the legend of Figure 4.

The MAPK family is a group of serine/threonine protein kinases, including ERK 1/2, p38MAPK, JNK/SAPK, and ERK 5, known to be involved in various cellular events such as survival/death, differentiation, and migration [19]. Initially, we examined the effects of 3′,5,5′,7-tetrahydroxyflavanone, luteolin, acacetin, or growth/neurotrophic factors (NGF or EGF) on the levels of phosphorylated MAPKs of PC12 cells. The cells were exposed to these substances at 100 uM, which was the concentration that had no effect on the expression of MAPK family proteins in PC12 cells. Luteolin, acacetin, NGF, and EGF significantly increased the phosphorylated levels of ERK 1/2 while 3′,5,5′,7-tetrahydroxyflavanone did not (Figure 4). Luteolin and acacetin also enhanced the levels of phosphorylated p38MAPK but 3′,5,5′,7-tetrahydroxyflavanone, NGF, and EGF did not (Figure 5). The levels of phosphorylated SAPK/JNK were substantially increased by NGF and slightly by luteolin and acacetin. However, 3′,5,5′,7-tetrahydroxyflavanone and EGF had no such effect (Figure 6).

### 3.6. Activation of Akt in PC12 Cells by the Isolated Components

**Figure 7:**
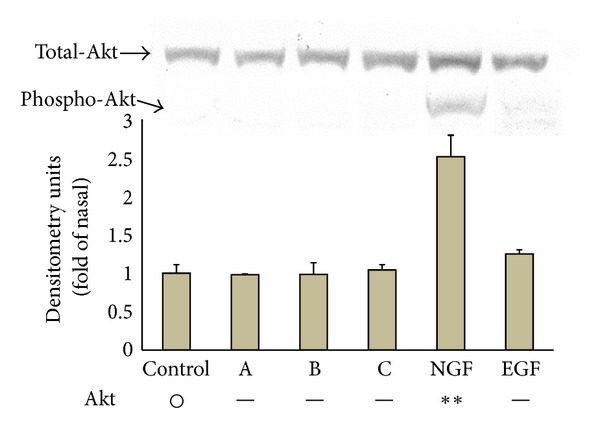
Effects of 3′,5,5′,7-tetrahydroxyflavanone (A), luteolin (B), acacetin (C), NGF, or EGF on the level of phosphorylated Akt of PC12 cells. Experimental procedures and methods for statistical analysis are the same as those of Figure 4.

It has been found that the activation of Akt as well as the MAPK family is required for neuronal differentiation [20]. Therefore, we examined the effects of the isolated components on the levels of phosphorylated Akt of PC12 cells. Although NGF significantly increased the level of phosphorylated Akt, the other substances, 3′,5,5′,7-tetrahydroxyflavanone, luteolin, acacetin, and EGF were not effective (Figure 7). From these results, luteolin and acacetin increased the levels of phosphorylated ERK 1/2, SAPK/JNK, and p38MAPK in PC12 cells but had no such effect on Akt. On the other hand, NGF elevated the levels of phosphorylated ERK 1/2, SAPK/JNK, and Akt but did not increase the level of phosphorylated p38MAPK. In addition, EGF significantly increased the phosphorylated level of ERK 1/2 and slightly increased that of Akt. Since the phosphorylated levels of neither MAPKs nor Akt in PC12 cells were unchanged by 3′,5,5′,7-tetrahydroxyflavanone, luteolin and acacetin were used for subsequent experiments.

### 3.7. Effects of Inhibitors on MAPK Activation by Luteolin and Acacetin

**Figure 8:**
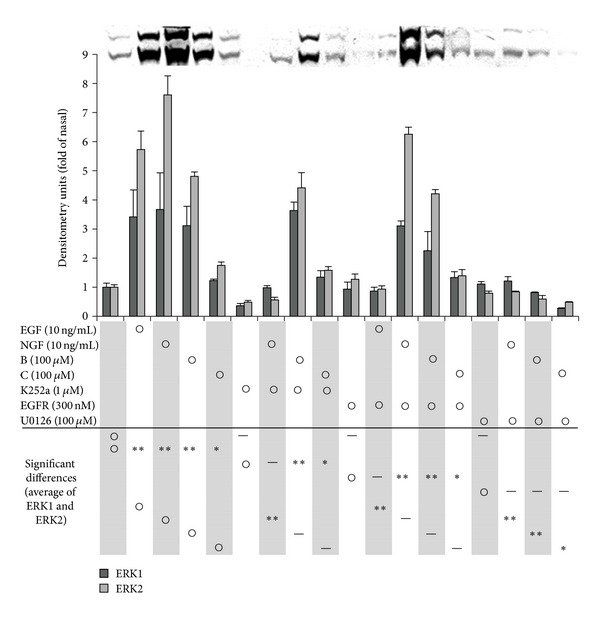
Effects of various inhibitors on the level of phosphorylated ERK 1/2 stimulated by luteolin (B), acacetin (D), NGF, or EGF in PC12 cells. PC12 cells were cultured in serum-containing medium for 2 days. The cells were preincubated with K252a, AG1478, SB203580 (1 *μ*M), or U0126 (10 *μ*M) in serum-containing medium for 3 h. They were then treated or not treated with B, D, NGF, or EGF for 10 min. The level of phosphorylated ERK 1/2 of each sample was evaluated by immunoblotting. The intensities of each band of ERK 1/2 were measured densitometrically by Image J. Values are expressed as the ratios of phospho-ERK 1/2 to total-ERK 1/2, and the mean ± SEM of the values of three separate experiments are shown. Significant differences of the values from the value of the corresponding control group were determined by ANOVA, followed by Dunnett's test. Control group is signified by ○. *: significance difference at *P* < 0.1; **: significant difference at *P* < 0.01, —: no significant difference.

We evaluated the effects of K252a (an inhibitor of tyrosine kinase of high-affinity NGF receptor Trk), AG1478 (an inhibitor of tyrosine kinase of EGF receptor), and U0126 (an inhibitor of MEK (ERK kinase)) on luteolin- or acacetin-induced phosphorylation of ERK 1/2 in PC12 cells (Figure 8). Phosphorylation was inhibited by U0126 but not by K252a or AG1478. Therefore, it was suggested that luteolin and acacetin activate MEK 1/2, resulting in enhanced activation/phosphorylation of ERK 1/2 (Figure 8). On the other hand, the phosphorylation of p38MAPK was inhibited by SB203580 (an inhibitor of p38MAPK phosphorylation) at a concentration of 1 *μ*M, while there was no such effect on ERK 1/2 (data not shown).

### 3.8. Neurite Outgrowth from PC12 Cells by Luteolin or Acacetin

**Figure 9:**
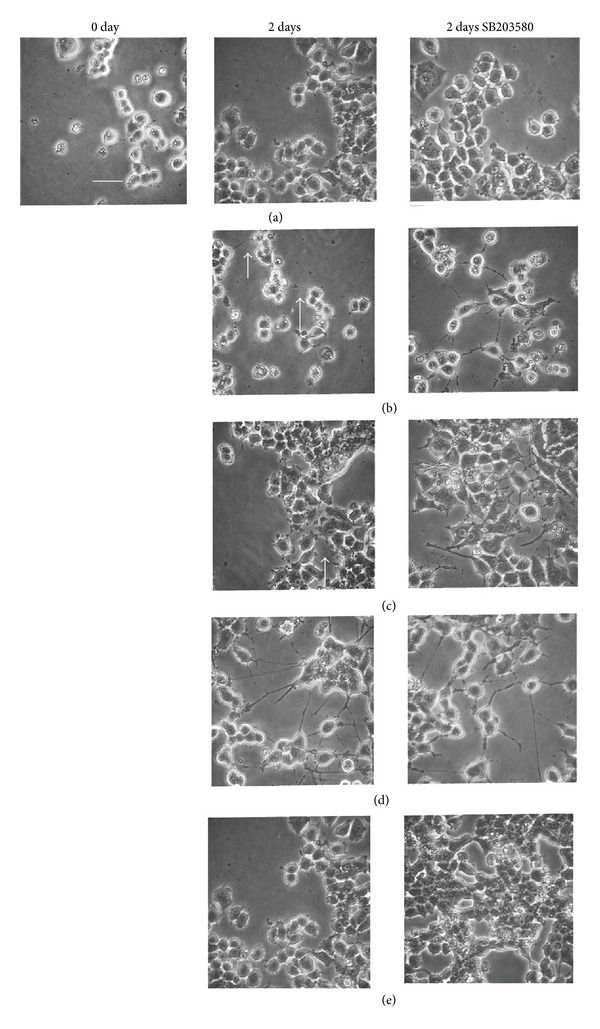
Effects of SB203580 on neurite outgrowth from cultured PC12 cells induced by control (a), luteolin (b), acacetin (c), NGF (d), or EGF (e). PC12 cells were cultured in serum-containing medium for 2 days. The cells were preincubated with or without SB203580 (10 *μ*M) for 3 h and then washed with PBS and treated with B, D, NGF, or EGF for 10 min. Cells were subsequently cultured for another 2 days in serum-free medium. Photographs were taken under phase-contrast observation at a magnification of 100x. Bar indicates 10 *μ*m. Arrows indicate weak neurites.

**Figure 10:**
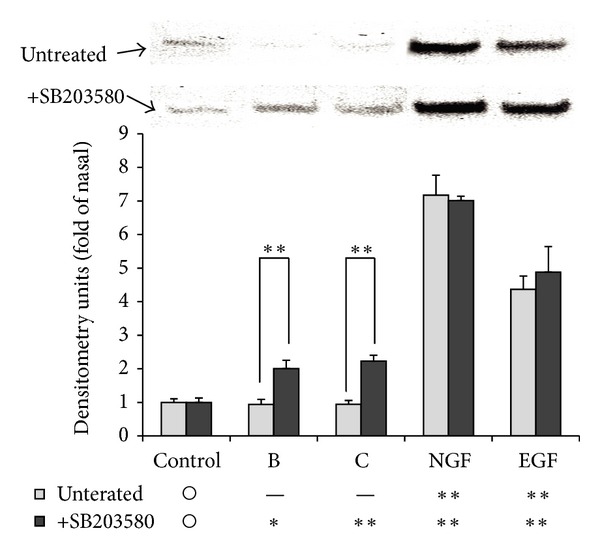
Effects of SB203580 on neurofilament-M expression of cultured PC12 cells induced by luteolin (B), acacetin (C), NGF, or EGF. PC12 cells were cultured and treated under the same conditions as shown in the legend of Figure 8. Cells were subsequently cultured for another 2 days in the serum-free medium. The expression of neurofilament-M of each sample was evaluated by immunoblotting. The intensity of each band was measured densitometrically by Image J. Values are expressed as the ratio of neurofilament-M to *β*-actin, and the means ± SEM of the values of three separate experiments are shown. Significant differences from the values of the corresponding control group were determined by ANOVA, followed by Dunnett's test. Control group is signified as ○. *: significance difference at *P* < 0.1; **: significant difference at *P* < 0.01; —: no significant difference. Arrows indicate slight neurite outgrowth.

PC12 cells were exposed to luteolin, acacetin, NGF, or EGF for 2 days in serum-free medium. Prominent neurite outgrowth was elicited by NGF and slight outgrowth by luteolin or acacetin (Figure 9). Interestingly, treatment with SB203580 enhanced acacetin- or luteolin-induced neurite outgrowth, suggesting the involvement of p38MAPK. Since neurite outgrowth has been found to correlate with the expression of neurofilament-M [21], we evaluated its expression in PC12 cells treated with the active molecules. NGF and EGF markedly increased the expression of neurofilament-M irrespective of SB203580 treatment (Figure 10). On the other hand, neither luteolin nor acacetin alone increased the expression of neurofilament-M; however, coadministration with SB203580 resulted in a significant increase in expression. These observations suggested that neurite outgrowth by both acacetin and luteolin was enhanced by the inhibition of p38MAPK phosphorylation.

## 4. Discussion

The well-known neurotrophic factor, NGF, acts on cultured PC12 cells and induces many biological events including neurite outgrowth for neuronal differentiation that gives rise to sympathetic neuron-like cells. These NGF actions require phosphorylation of the classical Ras/MAPK pathway. Based on this fact, extracts of Mottenohoka were tested and found to effectively enhance the level of phosphorylated ERK 1/2 of PC12 cells. The active components were isolated from the extracts and identified as 3′,5,5′,7-tetrahydroxyflavanone, acacetin, and luteolin. Acacetin and luteolin are known components of *Chrysanthemum morifolium Dendranthema × grandiflorum *[9–11]; however, the presence of 3′,5,5′,7-tetrahydroxyflavanone in chrysanthemum has not yet been clarified. We focused on luteolin and acacetin for further investigation because of the lack of a stimulatory effect of 3′,5,5′,7-tetrahydroxyflavanone on ERK 1/2 activation. Luteolin and acacetin promoted the phosphorylation of ERK 1/2, SAPK/JNK, and p38MAPK in PC12 cells at a concentration of 100 *μ*M but had no such effect on Akt. NGF, which stimulates neuronal differentiation of PC12 cells, facilitated the phosphorylation of ERK 1/2, SAPK/JNK, and Akt but not that of p38MAPK. EGF, which stimulates cell proliferation of PC12 cells, promoted significant phosphorylation of ERK 1/2 and slight phosphorylation of Akt. However, it did not promote the phosphorylation of SAPK/JNK and p38MAPK.

Luteolin and acacetin slightly but significantly stimulated neurite outgrowth, although this effect was much weaker than that of NGF (Figure 9). The intracellular signals between the active components and NGF differed: p38MAPK was activated by the active components but not by NGF, while the opposite was true for Akt. As the phosphorylation of Akt is considered to participate in cell growth arrest rather than neurite outgrowth [22], we focused on estimating whether neurite outgrowth is associated with the phosphorylation of p38MAPK. Treatment of PC12 cells with SB203580, an inhibitor of p38MAPK, significantly enhanced neurite outgrowth stimulated by luteolin or acacetin (Figure 9). Furthermore, the increase in neurofilament-M protein by pretreatment with SB203580 (Figure 10) confirmed that neurite outgrowth was enhanced by a decrease in the level of phosphorylated p38MAPK, because neurofilament-M is expressed in association with neurite outgrowth in PC12 cells [23]. Furthermore, epidermal growth factor (EGF), which enhances the level of phosphorylated ERK 1/2 but not that of phosphorylated p38MAPK, failed to induce neurite outgrowth. As SAPK/JNK was phosphorylated by NGF, the phosphorylation of SAPK/JNK as well as ERK 1/2 may be required for neurite outgrowth. However, pretreatment by a JNK inhibitor had no effect on neurite outgrowth stimulated by NGF (data not shown).

It has been suggested that sustained activation of ERK 1/2 is required for neuronal differentiation and neurite outgrowth in PC12 cells [24]. However, although both NGF and EGF promote phosphorylation of ERK 1/2 in PC12 cells, only NGF can induce neurite outgrowth. These differences in the biological actions of NGF and EGF on PC12 cells are due to the duration of a sustained level of ERK 1/2. NGF can induce long-lasting ERK 1/2 activation (phosphorylation) via activation of Crk and Src homology 2 (Shp-2) [25, 26]. These results suggest that the promotion or inhibition of p38MAPK phosphorylation is also related to neurite outgrowth. 

While the phosphorylation of p38MAPK is necessary for neural differentiation [27, 28], the phosphorylation of p38MAPK inhibits this process [29]. Thus, the results above suggested that the phosphorylation of p38MAPK could promote or inhibit neurite outgrowth depending on the experimental conditions.

K252a, an inhibitor of the Trk family of tyrosine kinases of high affinity neurotrophin receptors, is known to induce neurite outgrowth [30]. Since the expression of neurofilament-M (Figure 10) and the morphology of PC12 cells induced by NGF or EGF (Figure 9) were unchanged after SB203580 treatment, SB203580 alone is not thought to be involved in neurite outgrowth.

Luteolin- or acacetin-induced phosphorylation of ERK 1/2 was inhibited by U0126 (MEK phosphorylation inhibitor) treatment, leading to the assumption that the classical MAPK pathway was activated by luteolin or acacetin-stimulation. On the other hand, since the levels of luteolin- or acacetin-induced phosphorylated ERK 1/2 were unchanged by treatment with K252a or EGR receptor inhibitor, it can be deduced that luteolin or acacetin activates the classical MAPK cascade via receptors other than Trks or EGFR. Neurotrophic factors such as neurotrophins, including NGF, were predicted to be suitable therapeutic tools for some neurological disorders or as supplements for brain health. However, they are macromolecules that cannot cross the blood brain barrier and are unstable in the blood stream and tissues, thus studies have produced unsuccessful results. In the present study, we identified components in Mottenohoka that effectively activated Ras/MAPK(ERK 1/2) pathway of PC12 cells and led to neuritogenesis. The active entities were flavonoids, acacetin, and luteolin. A number of studies have dealt with flavonoids and it has been demonstrated that a component of ingested flavonoids moves into the plasma bound to albumin [25] and can pass through the blood-brain barrier [26].

In order to escape starvation during famines that have occurred regularly, plants that are not eaten in other regions are still cultivated and consumed in Yamagata Prefecture, Japan. Interestingly, it is possible that the flavonoids in such plants are useful in maintaining mental health because the number of suicides due to depression in Yamagata Prefecture is low compared with a neighboring prefecture.

Our results suggested that the inhibition of p38MAPK phosphorylation enhances neurite outgrowth stimulated by luteolin or acacetin. Thus, perhaps the combined use of previously examined natural components that promote neurite outgrowth and components that regulate p38MAPK phosphorylation could bring us a step closer to the commercialization of natural products for maintaining the health of the central nervous system.
